# *N-acetyltransferase 2 *(*NAT2*) gene polymorphisms in colon and lung cancer patients

**DOI:** 10.1186/1471-2350-7-58

**Published:** 2006-07-09

**Authors:** Juergen Borlak, Stella Marie Reamon-Buettner

**Affiliations:** 1 Drug Research and Medical Biotechnology, Fraunhofer Institute of Toxicology and Experimental Medicine, Nikolai-Fuchs-Strasse 1, 30625 Hannover, Germany; 2 Chair in Pharmaco-and Toxicogenomics, Center of Pharmacology and Toxicology, Medical School Hannover, Carl-Neuberg-Strasse 1, 30625 Hannover, Germany

## Abstract

**Background:**

*N*-acetyltransferase 2 (NAT2) metabolizes arylamines and hydrazines moeities found in many therapeutic drugs, chemicals and carcinogens. The gene encoding NAT2 is polymorphic, thus resulting in rapid or slow acetylator phenotypes. The acetylator status may, therefore, predispose drug-induced toxicities and cancer risks, such as bladder, colon and lung cancer. Indeed, some studies demonstrate a positive association between NAT2 rapid acetylator phenotype and colon cancer, but results are inconsistent. The role of NAT2 acetylation status in lung cancer is likewise unclear, in which both the rapid and slow acetylator genotypes have been associated with disease.

**Methods:**

We investigated three genetic variations, c.481C>T, c.590G>A (p.R197Q) and c.857G>A (p.G286E), of the *NAT2 *gene, which are known to result in a slow acetylator phenotype. Using validated PCR-RFLP assays, we genotyped 243 healthy unrelated Caucasian control subjects, 92 colon and 67 lung cancer patients for these genetic variations. As there is a recent meta-analysis of *NAT2 *studies on colon cancer (unlike in lung cancer), we have also undertaken a systematic review of *NAT2 *studies on lung cancer, and we incorporated our results in a meta-analysis consisting of 16 studies, 3,865 lung cancer patients and 6,077 control subjects.

**Results:**

We did not obtain statistically significant differences in *NAT2 *allele and genotype frequencies in colon cancer patients and control group. Certain genotypes, however, such as [c.590AA+c.857GA] and [c.590GA+c.857GA] were absent among the colon cancer patients. Similarly, allele frequencies in lung cancer patients and controls did not differ significantly. Nevertheless, there was a significant increase of genotypes [c.590GA] and [c.481CT+c.590GA], but absence of homozygous c.590AA and [c.590AA+c.857GA] in the lung cancer group. Meta-analysis of 16 *NAT2 *studies on lung cancer did not evidence an overall association of the rapid or slow acetylator status to lung cancer. Similarly, the summary odds ratios obtained with stratified meta-analysis based on ethnicity, and smoking status were not significant.

**Conclusion:**

Our study failed to show an overall association of *NAT2 *genotypes to either colon or lung cancer risk.

## Background

***N***-acetyltransferases (NAT; E.C.2.3.1.5) catalyze the metabolism of various aromatic amine drugs and carcinogens. Sequence variations in the human *NAT1 *(MIM# 108345) and *NAT2 *(MIM# 243400) have been associated with drug-induced toxicities and disease (see reviews, [1-4]. Such sequence variations result in the production of NAT proteins with variable enzyme activity or stability, leading to slow or rapid acetylation. Indeed, an association with either slow or rapid acetylation has been reported for different cancers, systemic lupus erythematosis, diabetes, and Alzheimer's disease [3]. Specifically, the type of acetylator status may predispose a person to a particular cancer risk [5]. For instance, for cancers in which *N*-acetylation is a detoxification step such as aromatic amine-related urinary bladder, NAT2 slow acetylator phenotype seems at higher risk. For cancers in which *N*-acetylation is negligible and *O*-acetylation is an activation step such as heterocyclic amine-related colon cancer, NAT2 rapid acetylator phenotype is at higher risk. Several studies showed an association between slow acetylator phenotype and urinary bladder cancer risk, as well as rapid acetylator phenotype and colon cancer risk (reviewed in [6]). A meta-analysis of 20 case-control studies showed, however, that NAT2 rapid acetylation status has no specific effect on the risk of colon cancer [7]. These conflicting results may be clarified by a careful determination of *NAT2 *genotypes in colon cancer patients.

So far, the role of NAT2 acetylation status in lung cancer is unclear, in which both the rapid and slow acetylator genotypes have been implicated in disease. Presumably, exposure of lungs to various environmental carcinogens and cigarette smoke, as well as ethnic and genetic differences, may influence results. We found at least 15 case-control studies (five of which are published in 2005) on *NAT2 *variants and lung cancer risk in different ethnic groups and exposure variables, such as cigarette smoke and asbestos [8-22]. Most studies obtained no overall association of *NAT2 *acetylator genotypes to the development of lung cancer, but specific risks were detected. For instance, there was an increased risk with homozygous NAT2*4 genotype, especially if gender, age and smoking factors are considered [9]; with homozygous c.341C+481T+803G and c.590A alleles [8]; with slow acetylator genotype in adenocarcinoma in patients < 65 years old [10]; or with slow acetylator in non-operable lung cancer, younger age, and lower smoking dose [14]. Furthermore, a significant interaction between *NAT2 *genotype and pack-years of smoking was found, in which rapid acetylator was protective in non-smokers, but a risk factor in heavy smoker [17].

The human *NAT2 *gene is located on chromosome 8p22 [23,24] and encodes a 290-amino-acid protein. The gene is polymorphic and 36 alleles have been so far described [25]. Many of the NAT2* alleles share sequence variations, and not all sequence variations would lead to change in the enzyme activity of the coded protein. To determine *NAT2 *genotypes in our colon and lung cancer patients and control population of healthy individuals, we investigated three sequence variations reported to result in impaired acetylation. We did not only analyzed overall rapid or slow acetylator genotypes, but paid particular attention to individual *NAT2 *genotypes which may confer susceptibility to colon and lung cancer. We have also undertaken a systematic review of *NAT2 *studies on lung cancer and we incorporated our present results in a meta-analysis consisting of 16 studies, 3,865 patients and 6,077 control subjects.

## Methods

Informed consent blood samples were kindly provided by Dr. C.A.D Smith of the Imperial Cancer Research Fund Laboratory of the Ninewell's Hospital in Dundee, U.K. In addition, JB has obtained approval to conduct genetic studies involving human materials from the Medical School of Hannover, Germany. All blood samples were obtained from randomly selected, unrelated Caucasian individuals. Using standard PCR-RFLP assay protocols, we employed the restriction enzymes *Kpn*I, *Taq*I and *Bam*HI to distinguish *NAT2 *variations c.481C>T (p.161L, dbSNP rs1799929), c.590G>A (p.R197Q, dbSNP rs1799930) and c.857G>A (p.G286E, dbSNP rs1799931), respectively.

On the basis of these *NAT2 *variations, we genotyped 243 healthy unrelated Caucasian control subjects and 92 colon and 67 lung cancer patients. The reference allele (NAT2*4) contains all three restriction sites, thus the identification of homo- and heterozygous carriers could easily be ascertained. In accordance with the human *NAT2 *nomenclature [25], allele NAT2*4 refers to *NAT2 *reference sequence (Genbank accession X14672). The NAT2*4 allele acts dominantly to result in rapid acetylation, and the presence of c.481C>T, c.590G>A, c.857G>A would lead to slow acetylation [26]. The acetylation status for the synonymous variation c.481C>T is not clear as this is also associated with allele NAT2*12C (c.481C>T + c.803A>G) which is actually a rapid allele [1]. For the determination of acetylator status, we classified those possessing at least two mutant alleles as slow acetylators. Except for c.590G>A in lung cancer, genetic variations were in Hardy-Weinberg equilibrium in colon and lung cancer patients, as well as in controls.

Differences in allele and genotype frequency distributions in cases and controls were determined by χ^2^-tests, with significance at p ≤ 0.05. For the computation of percentages, odds ratios (OR) with 95% confidence interval and χ^2 ^tests, we used the statistical package SPSS 14.0. The genotype NAT2*4/*4 [c. 481CC + c.590 GG + c.857 GG] was used as reference and ORs were calculated with respect to this reference genotype. For the systematic review and meta-analysis, we performed a search on Medline (PubMed) and Embase using the keywords 'NAT2' and 'lung cancer'. We also searched references of retrieved studies as well as reviews on NAT2. Only studies published in English were retrieved and included in the meta-analysis.

Meta-analysis was performed using the package rmeta of the R-Project [27]. The Mantel-Haenszel procedure (fixed effects model) and the DerSimonian-Laird procedure (random effects model) were applied to analyze the odds ratios of the studies, the summary OR, and Woolf's test for heterogeneity. Also the 95% confidence intervals were calculated for the individual and the summary odds ratios.

## Results

### Colon cancer

We observed the reference allele NAT2*4 [c.481C+c.590G+c.857G] in 22.3% and 28.0% for control and colon cancer patients, respectively. Similarly, the frequency of the c.481C>T variant was 48.9% and 37.5%; the c.590G>A was 27% and 34.2%; and the c.857G>A was 1.7% and 1.6% in the control and colon cancer populations, respectively. The difference in these allele frequencies as well as those of individual genotype frequencies (Table 1) did not reach statistical significance. Nevertheless, the genotypes [c.590AA+c.857GA] as well as [c.590GA+c.857GA] were not detected among the colon cancer patients, as compared with controls. The distribution of overall genotypes was 43.5% (40/92) rapid and 56.5% (52/92) slow acetylator in the colon cancer patients, and 37.4% (91/243) rapid and 62.6% (152/243) slow acetylator in the controls, and the odds ratio (OR) obtained for rapid acetylator status in cases vs. controls was 1.29 (95% CI 0.79–2.10, p > 0.05), which was not significant.

**Table 1:** *NAT2 *genotypes in controls and colon cancer patients

Genotypes			Deduced phenotypes	Controls	Colon cancer	Total	Odds ratios (95% confidence interval)^2^
			
c.481 C>T	c.590 G>A	c.857 G>A		*n *= 243	*n *= 92	*n *= 335	
CC	GG	GG	rapid	5.4% (13)	9.8% (9)	6.6% (22)	reference
CT			rapid	23.0% (56)	18.5% (17)	21.8% (73)	0.44 (0.16–1.20)
	GA		rapid	8.6% (21)	13.0% (12)	9.9% (33)	0.83 (0.27–2.49)
		GA	rapid	0.4% (1)	2.2% (2)	0.9% (3)	2.89 (0.23–36.87)
TT			slow^1^	22.2% (54)	13.0% (12)	19.7% (66)	0.32 (0.11–0.92)**
	AA		slow	9.5% (23)	13.0% (12)	10.4% (35)	0.75 (0.25–2.26)
		AA	slow	0	0	0	
	AA	GA	slow	4.1% (10)	0	3.0% (10)	
CT	GA		slow	23.9% (58)	29.3% (27)	25.4% (85)	0.67 (0.26–1.77)
CT		GA	slow	2.5% (6)	1.0% (1)	2.1% (7)	0.24 (0.03–2.36)
	GA	GA	slow	0.4% (1)	0	0.3% (1)	

^1 ^c.481C>T is a silent mutation associated also with NAT*12C (c.481C>T, c.803A>G) which is a rapid allele (see Hein et al. 2000, [1])

^2 ^For the calculation of ORs for individual genotypes, NAT2*4/*4 [c. 481CC + c.590 GG + c.857 GG] was used as reference and all corresponding odds ratios were determined with respect to this reference.

** significant.

For the calculation of ORs for individual genotypes, NAT2*4/*4 [c. 481CC + c.590 GG + c.857 GG] was used as reference and all corresponding odds ratios were determined with respect to this reference (Table 1). By doing so, the ORs measure the chance for increased risk of colon cancer as compared to the reference, if a certain genotype is present. This analysis did not find statistically significant ORs, except for the genotype c.481 TT at p = 0.03 (see Table 1).

### Lung cancer

We obtained for the reference allele NAT2*4 [c.481C + c.590G + c.857G] in 22.3% and 24.6% for control and lung cancer patients, respectively. Similarly, the frequency of the c.481C>T variant was 48.9% and 41.3%; the c.590G>A was 27.0% and 31.5%; and the c.857G>A was 1.7% and 2.9% in the control and lung cancer populations, respectively. The difference in allele frequencies between lung cancer patients and controls was not statistically significant. However, carriers of genotypes [c.590AA], [c.590AA+c.857GA] and [c.857GA], were not detected within the lung cancer group (Table 2). This may or may not be the result of the small population size, *n *= 67 patients. However, within the control group, the total frequencies of these genotypes accounted for 14% or 34 individuals from 243. Furthermore, we did not detect homozygous c.857AA (also in colon cancer patients and controls), which is rare among Caucasians [28].

**Table 2:** *NAT2 *genotypes in controls and lung cancer patients

Genotypes			Deduced phenotypes	Controls	Lung cancer	Total	Odds ratios (95% confidence interval)^2^
			
c.481 C>T	c.590 G>A	c.857 G>A		*n *= 243	*n *= 67	*n *= 310	
CC	GG	GG	rapid	5.3% (13)	7.5% (5)	5.8% (18)	reference
CT			rapid	23.0% (56)	13.4% (9)	21.0% (65)	0.42 (0.12–1.46)
	GA		rapid	8.6% (21)	20.9% (14)	11.3% (35)	1.73 (0.50–5.95)
		GA	rapid	0.4% (1)	0	0.3% (1)	
TT			slow^1^	22.2% (54)	13.4% (9)	20.3% (63)	0.43 (0.12–1.51)
	AA		slow	9.5% (23)	0	7.4% (23)	
		AA	slow	0	0	0	
	AA	GA	slow	4.1% (10)	0	3.2% (10)	
CT	GA		slow	23.9% (58)	38.8% (26)	27.1% (84)	1.17 (0.38–3.60)
CT		GA	slow	2.5% (6)	4.5% (3)	2.8% (9)	1.30 (0.23–7.31)
	GA	GA	slow	0.4% (1)	1.4% (1)	0.6% (2)	2.60 (0.14–50.04)

^1^c.481C>T is a silent mutation associated also with NAT*12C (c.481C>T+ c.803A>G) which is a rapid allele (see Hein et al. 2000, [1])

^2 ^For the calculation of ORs for individual genotypes, NAT2*4/*4 [c. 481CC + c.590 GG + c.857 GG] was used as reference and all corresponding odds ratios were determined with respect to this reference.

The distribution of overall genotypes was 41.8% (28/67) rapid and 58.2% (39/67) slow acetylator in the lung cancer patients, and 37.4% (91/243) rapid and 62.6% (152/243) slow acetylator in the control group, and the odds ratio (OR) we obtained for slow acetylator status in cases vs. controls was 0.83 (95% CI, 0.48–1.45, p > 0.05), which was not significant. The genotype NAT2*4/*4 [c. 481CC + c.590 GG + c.857 GG] was also used as reference for the calculation of individual ORs. In the same manner, the ORs measure the chance for increased risk of lung cancer as compared to the reference, if a certain genotype is present. The ORs obtained for individual genotypes (Table 2) were not significant.

Many studies have been undertaken on *NAT2 *variants and lung cancer risk, of which five recently published (see Background). In contrast to colon cancer, where a meta-analysis on 20 studies has been undertaken [7], none is available for lung cancer. We summarized 15 published studies (Table 3, see additional file 1) and carried out a meta-analysis on 16 studies (including our own) to a total of 3,865 lung cancer patients and 6,077 control subjects. These published case-control studies were mostly hospital-based and original studies, except Sorensen et al. [18] which was population-based and Skuladottir et al. [20], which was based on pooled data from three studies of Danish and Norwegian Caucasians. Basically, studies were carried out on Caucasians, except three on Asians (2 Chinese, 1 Japanese). Samples size of lung cancer cases ranged from 108–150 (4 studies), 153–185 (5 studies), 205–282 (3 studies), 320–392 (2 studies) and 1,115 (1 study). Average % males in 13 studies was 65%, (range 0–100%) in cases, while in controls, 55% (0–100%). Except for three studies [12,13,22], on which emphasis was on never smoking women, studies were undertaken on male smokers; for example 7 studies with 76–100% males and 89–100% smokers in lung cancer cases [8,9,11,14,16,19,21]. As regards histology of lung cancer cases, adenocarcinoma ranged from 19–78% in 10 studies, and in 4 studies more than 50% [10,12,13,22]. Squamous cell carcinoma ranged from 20–65% in 12 studies, and in 7 studies, average was about 50%, range 41–65% [8,10,11,14,16,19,21]. Next frequent types were small cell (5–35%) in 8 studies, large cell carcinoma in 4 studies (0.04%–18%).

**Figure 1:**
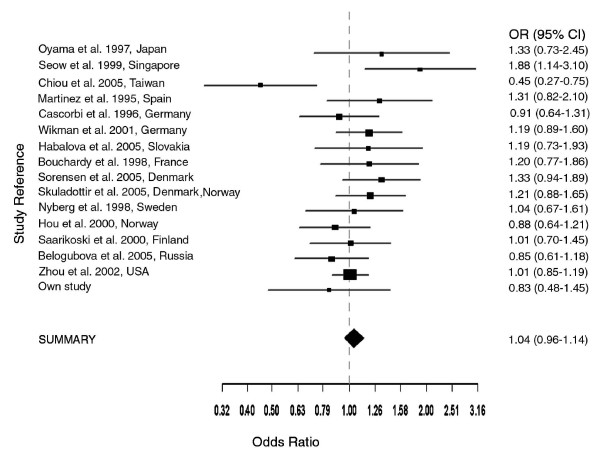
**Meta-analysis (Mantel-Haenszel, fixed effects) on 16 case-control association studies on NAT2 slow acetylator status and lung cancer**. Test for heterogeneity χ^2 ^(df 15) = 25.58 (p = 0.0426). The summary odds ratio (OR = 1.04, 95% CI, 0.96–1.14) is not significant. Meta-analysis (DerSimonian-Laird, random effects) is similar, with summary OR (1.05, 95% CI 0.93–1.19, estimated random effects variance = 0.02), likewise not significant. The confidence interval for each study is given by a horizontal line, and the point estimate is given by a square whose height is inversely proportional to the standard error of the estimate. The summary odds ratio is drawn as a diamond with horizontal limits at the confidence limits and width inversely proportional to its standard error.

Results of meta-analysis showed that except for the two studies on Chinese populations (Seow et al. [13], Chiou et al. [22]) none of the ORs were significant (Fig. 1). For the meta-analysis of slow acetylators (2,149/3,865 lung cancer patients vs. 3,276/6,077 control subjects), we obtained a summary OR value of 1.04 (95% CI 0.96–1.14) which was not significant (Fig. 1). This meta-analysis was based on a fixed effects model (Mantel-Haenszel) which assumes that studies use identical methods, patients and measurements, and differences are only due to within study differences. Since the test for heterogeneity, however, was significant (χ^2 ^(df 15) = 25.58, p-value 0.0426), we also carried out a meta-analysis using a random effects model (DerSimonian-Laird) which considers both between-study and within-study variability. The individual odds ratios for the 16 studies did not change, but there was a small difference in the summary OR (1.05, 95% CI 0.93–1.19, and random effects variance = 0.02) as compared to that obtained from the fixed effects model. Nonetheless, the summary OR obtained from the random effects model was also not significant. Furthermore, we also performed Egger's test to assess potential publication bias, which results from non-publication of small studies with negative results. The Egger's test showed no evidence of publication bias (p = 0.75).

**Figure 2:**
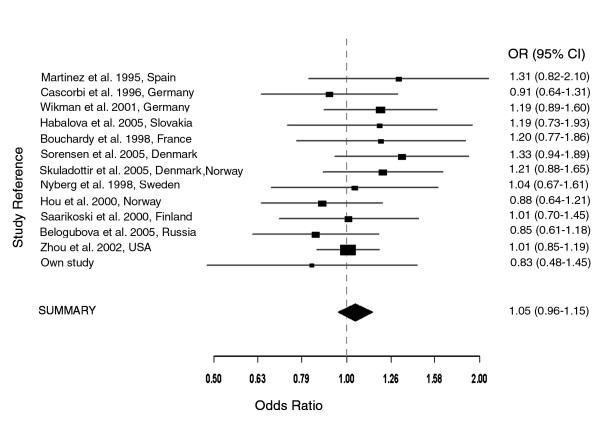
**Meta-analysis (Mantel-Haenszel, fixed effects) on 13 case-control association studies on NAT2 slow acetylator status and lung cancer – Caucasians**. Test for heterogeneity χ^2 ^(df 12) = 9.02 (p = 0.7012). The summary odds ratio (OR = 1.05, 95% CI, (0.96–1.15) is not significant.

**Figure 3:**
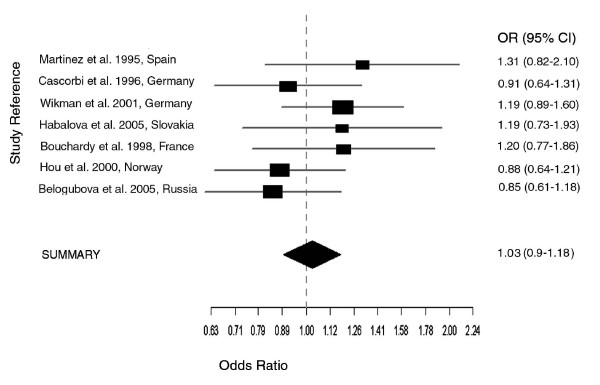
**Meta-analysis (Mantel-Haenszel, fixed effects) on 7 case-control association studies on NAT2 slow acetylator status and lung cancer – Caucasians, male smokers**. The summary odds ratio (OR = 1.03 (95% CI, 0.9–1.18) is not significant.

Since the effect of NAT2 on lung cancer may be influenced by ethnicity and smoking status, we carried out a stratified meta-analysis of studies based on Asians, Caucasians and, male Caucasian smokers. Although the test for heterogeneity was statistically significant only in the Asian studies (p = 3e-04), results from both fixed and random effects models were compared to determine the extent of variations. The meta-analysis on three studies on Asian populations (Oyama et al. [10], Seow et al. [13] Chiou et al. [22]) gave a summary OR of 1 (95% CI 0.74–1.35) for fixed effects model, while for random effects model, an OR of 1.04 (95% CI, 0.43–2.53) and an estimated random effects variance of 0.54. For the meta-analysis of 13 studies on Caucasian populations, the summary OR obtained from both fixed and random effects models was identical, e.g. 1.05 (95% CI, 0.96–1.15) (see Fig. 3). In similar manner, the summary OR for both models was 1.03 (95% CI, 0.9–1.18) in the meta-analysis of studies on male Caucasian smokers. Nonetheless, summary ORs from both fixed and random effects models were not significant, and results from Egger's test did not indicate publication bias (p > 0.05).

## Discussion

Genetic analysis of three *NAT2 *genetic variations (c.481C>T, c.590G>A and c.857G>A) in 92 colon cancer patients and 243 controls, both of unrelated Caucasians, failed to show statistically significant overall association of acetylator genotypes, in particular rapid acetylator genotypes, to colon cancer risk. But certain *NAT2 *individual genotype may be associated with colon cancer risk, such as [c.481 TT], which gave statistically significant ORs, when calculated in relation to NAT2*4/*4 as reference genotype. This genotype showed an inverse association to colon cancer risk and therefore may lend protection against the disease. However, as mentioned in Methods, the acetylation status for the synonymous variation c.481C>T is not clear, because it is also associated with NAT2*12C (c.481C>T+c.803A>G) which is a rapid allele [1].

There is published evidence to suggest an overrepresentation of rapid acetylators in patients diagnosed with colorectal carcinoma [29] and some studies are supportive for a relationship between intake of meat and risk of colon cancer in certain *NAT *genotype constellation. Indeed, the study of Kiss et al. [30] provided evidence for carrier of the rapid acetylator alleles to produce higher levels of DNA strand breaks in exfoliated colorectal mucosa cells following a two-day "high meat" diet. Thus, the genotypes of *NAT1 *and *NAT2 *coding for the rapid acetylator phenotype carry a higher risk for colorectal cancer [31]. This association was also confirmed in a Chinese population with diagnosed colorectal carcinoma [32]. By contrast, a meta-analysis of 20 case-control studies on NAT2 acetylation status [7], and an investigation into an interaction between NAT2 and heterocyclic amines derived from meat do not provide evidence for rapid acetylators to be at higher risk for developing colon cancer [33]. Finally, there is interindividual variation in *NAT1 *and *NAT2 *expression in the colon which could affect response to exposure to specific NAT substrates including dietary carcinogens [34].

Similar to colon cancer, the difference in allele frequencies after genotyping the same *NAT2 *gene variations in 67 lung cancer patients and 243 controls was not statistically significant. This may or may not have resulted from the small sample size and for not taking into account, certain exposure variables which may influence results. However, with the exception of two studies on Chinese populations, none of the 15 reviewed studies found an overall association of *NAT2 *acetylation genotypes to lung cancer risk (Table 3, see additional file 1). These findings were corroborated by the individual odds ratios obtained in the meta-analysis (see Fig. 1). So far, results of all carried out meta-analyses did not evidence overall association of *NAT2 *acetylation genotypes.

Indeed, even in a very big study subjects of 1,115 lung cancer cases, and in which age, gender, smoking status and pack years of smoking were considered, no overall relationship between *NAT2 *genotypes and lung cancer risk was obtained [17]. Similarly, the use of 'comparison of extremes approach' in which the distribution of *NAT2 *genotypes in lung cancer patients were compared not only to the population controls, but also to elderly tumor-free smokers and non-smokers, did not find an association even if smoking history, gender, age or lung cancer histology [19]. Furthermore, results of the meta-analysis also failed to demonstrate an overall significant association of *NAT2 *acetylation genotypes in lung cancer.

Closer examination, however, of the different studies on NAT2 acetylation status and lung cancer risk, showed lack of uniformity in the design, analysis, reporting and exposure variables (e.g. smoke, asbestos) tested. These factors may have led to discrepancy over results. Although most studies found no overall association, specific risks were observed. Indeed, significant associations of *NAT2 *acetylation genotypes to lung cancer risk were obtained from non-smoking women Chinese women [13,22], and not on male smokers which were mainly the case subjects in several studies (for example, 7 studies with 76–100% males and 89–100% smokers in lung cancer cases [8,9,11,14,16,19,21]). Yet different results have been observed on non-smoking Chinese women; e.g. either the slow acetylator genotype was associated with increased risk of lung cancer among non-smoking Chinese women in Singapore [13], or the rapid acetylator genotype was associated with an increase risk of lung cancer among never smoking Chinese women in Taiwan [22].

There are also other factors which may modify susceptibility to lung cancer. For instance, *NAT2 *genotypes may provide a risk to lung cancer when combined with other genes. Individuals, with combined *NAT1 *rapid and *NAT2 *slow genotype seemed to have significantly elevated adenocarcinoma risk [16], or the *NAT2 *slow genotype when combined with the *GSTM1 *null genotype may confer increased susceptibility to adduct formation, gene mutation and lung cancer when the smoking dose is low [35]. The combination *NAT2 *slow-*CYP1A1 *rapid acetylator were at highest risk for lung adenocarcinoma in non-smoking females [36] or the *NAT2*-*CYP1A1 *rapid acetylators may also predispose higher risk to lung cancer in female never smokers [22].

## Conclusion

Our study failed to show an overall association of *NAT2 *genotypes to either colon or lung cancer risk, but results are underpowered due to small sample size and to other factors not considered in our analysis. As certain *NAT2 *genotypes may render susceptibility to cancer risk, perhaps a collaborative effort where specific *NAT2 *genotypes are investigated with uniform study design, analysis, reporting and exposure variables in different ethnic groups is needed.

## Competing interests

The author(s) declare that they have no competing interests.

## Authors' contributions

JB was responsible for the conception and design, acquisition and analysis of data, interpretation of results and writing of the manuscript, and have given final approval of the version to be published. SMRB participated in the conception and design, acquisition and analysis of data, interpretation of results and writing of the manuscript.

## Pre-publication history

The pre-publication history for this paper can be accessed here:



## Supplementary Material

Additional file 1 **Table 3-Summary of studies on *NAT2 *acetylation genotypes and lung cancer risk**. Fifteen case-control studies on *NAT2 *and lung cancer risk carried out from 1995 to 2005 summarized by authors/country, ethnicity of study subjects, % slow acetylators in cases and controls, *NAT2 *variations analyzed, methods, and results of association analysis. Click here for file
