# Supplementary material for: N-acetyltransferase 2 (NAT2) gene polymorphisms in colon and lung cancer patients
Source: BMC Med Genet. 2006 Jul 9;7:58. doi: 10.1186/1471-2350-7-58 (PMC1533812; doi:10.1186/1471-2350-7-58)
Supplement: Additional file 1 — Table 3-Summary of studies on NAT2 acetylation genotypes and lung cancer risk. Fifteen case-control studies on NAT2 and lung cancer risk carried out from 1995 to 2005 summarized by authors/country, ethnicity of study subjects, % slow acetylators in cases and controls, NAT2 variations analyzed, methods, and results of association analysis. [file 1471-2350-7-58-S1.doc]

## Table 3- Summary of studies on NAT2 acetylation genotypes and lung cancer risk

| **Authors,**  **Country** | **Ethnicity** | **Cases** | **% Slow**  **acetylators** | **Controls** | **% Slow**  **acetylators** | ***NAT2***  **variations**  **(nt position)** | **Methods** | **Association**  **(Yes/No)** |
| --- | --- | --- | --- | --- | --- | --- | --- | --- |
| Martinez et al.  1995, Spain | Spanish  Caucasians | *n*=108; 95% males; 61±11(31-86 yr);  95% smokers; 25 adenocarcinoma,  22 small cell, 54 squamous cell,  7 others; San Carlos University Madrid, 1991-1993 | 70/108  (64.8%) | *n*=243; 43% males; 25±18 (18-81 yr);  San Carlos University Madrid and  Dept of Pharmacology,  Medical School,Badajoz; 1991-1993 | 142/243  (58.4%) | 191, 282, 341, 590, 803 | blood samples;  allele-specific PCR; OR 95% CI, χ^2^-test, Fisher's exact test | No, but increased risk with homozygous  341C+481T+803G  and 590A alleles |
|  |  |  |  |  |  |  |  |  |
| Cascorbi et al.  1996, Germany | German  Caucasians | *n*= 155; 80% males, 56 (37-87 yr);  96% smokers; 50 squamous cell,  20 small cell, 28 large cell,  33 adenocarcinoma, 24 mixed cell;  Lungenklinik Heckeshorn, 1991-1994 | 87/155  (56.1%) | control 1: *n*= 310; 80% males; 65 (27-87yr); 68% smokers; reference patients with non-malignant disease from various hospitals Berlin  control 2: *n*= 278; 72% males;  30 (20-82 yr); healthy volunteers | control 1:  181/310 (58.4%)  control 2:  162/278  (58.3%) | 191, 282, 341, 481, 590, 803, 857 | blood samples,  PCR-RFLP; sequencing,  caffeine test;  Fisher's exact test,  OR 95% CI, logistic regression  analysis (gender, age, smoking),  BMDP program | No, but increased risk with homozygous *NAT2*4*  especially if gender,  age, and smoking  considered |
|  |  |  |  |  |  |  |  |  |
| Oyama et al.  1997, Japan | Japanese  (Asians) | *n*=124; 73% males; 66(32-83 yr); 73 adenocarcinoma, 51 squamous cell; underwent surgical resection 1990-1994 | 17/124  (13.7%) | *n*=376; 91% males; 36 (20-71 yr);  factory workers, Kyushu | 40/376  (10.6%) | 191, 481, 590, 857 | tumors and normal lung tissue for patients, blood samples controls,  PCR-RFLP; χ^2^-test, t-test,  Mantel-Haenszel relative risk (RR) | No, but increased RR with slow acetylators adenocarcinoma  < 65 yr |
|  |  |  |  |  |  |  |  |  |
| Bouchardy et al. 1998, Switzerland, Finland, France | French  Caucasians | *n*=150; 93% males; 58.4 yr; regular  smokers; 98 squamous cell, 52 small  cell carcinoma; from various hospitals  (9 of 10 Paris) 1988-1992;  19% occupational asbestos exposure | 86/150  (57.3%) | *n*=172; 95% males; 55.0 yr; regular smokers; patients nonmalignant disease; 7% occupational asbestos exposure | 91/172  (53.0%) | 481, 590, 857 | blood samples;  PCR-RFLP; OR 95% CI  unconditional multivariate logistic  regression analysis, ORs  adjusted by age, gender, smoking, occupational exposure | No, even if age, gender, smoking and occupational exposure considered;  No combined  *NAT1-NAT2* risk |
|  |  |  |  |  |  |  |  |  |
| Nyberg et al.  1998, Sweden | Swedish  Caucasians | *n*=185; 25.4% males; 30 to ≥80 yr;  48 % never smokers; 94  adenocarcinoma, 47 squamous cell,  9 small cell, 7 large cell, 26 carcinoid;  10 others; three hospitals Stockholm,  1992-1995 | 113/183  (61.7%) | *n*=164; 28.7% males; 30 to ≥80 yr;  48 % never smokers,  Stockholm population, 1995 | 96/158  (60.8%) | 341,481,590, 803, 857 | blood samples;  PCR-RFLP; OR 95% CI unconditional logistic regression analysis | No, but increased risk slow acetylators never-smokers, increased risk rapid acetylators smokers;  High risk  *GSTM1*^+^ -*NAT2* slow  in never-smokers |
|  |  |  |  |  |  |  |  |  |
| Seow et al.  1999, Singapore | Chinese  (Asians) | *n*=153; 0% males; 65.2±12.6 yr;  60% non-smokers; 86 adenocarcinoma, 31 squamous cell, 15 small cell, 21 large cell;  three hospitals Singapore 1997-1998 | 60/153  (39.2%) | *n*=141; 0% males; 63.4±12.4 yr;  89 % non-smokers; patients from  same hospitals with no cancer/chronic respiratory condition | 36/141  (25.5%) | 481, 590, 857 | blood samples;  allele specific PCR; OR 95% CI, adjusted ORs logistic regression analysis,  SPSSWIN 8.0 | Yes, increased risk with  slow acetylators in women  non-smokers |
|  |  |  |  |  |  |  |  |  |
| Saarikoski et al.  2000, Finland,  France | Finnish  Caucasians | *n*= 205; Helsinki University Central  Hospital, 1988-1997; operable lung  cancer, asbestos-exposed | 102/195  (52.3%) | *n*=294; population control | 152/292  (52.1%) |  | OR, 95% CI, two-sided  Mantel-Haenszel method | No, but increased risk in  high asbestos-exposure |
|  |  |  |  |  |  |  |  |  |
| Hou et al. 2000, Sweden, Norway | Norwegian  Caucasians | *n*= 282; 100% males; 63(30-85yr);  97% (244/252) smokers; 147  non-operable; 135 operable lung  cancer; 129 squamous cell, 41 small  cell, 54 adenocarcinoma, 31 large cell;  three hospitals (2 Oslo, 1Bergen) | 169/281  (60.1%) | *n*=375; 100% males; 50(23-89 yr;  56% smokers; current or previous  workers; arbitrarily selected from  several companies, Norway | 237/375  (63.2%) | 341, 481,  590, 857 | blood samples;  PCR-RFLP; χ^2^-test, OR Cornfield 95% CI or Woolf 95% CI for small size, Mantel-Haenszel heterogeneity test,  logistic regression for interactions | No, but increased risk with slow acetylator, in non-operable lung cancer, younger age, and lower smoking dose |
|  |  |  |  |  |  |  |  |  |
| Wikman et al.  2001, Germany | German  Caucasians | *n*=392; 76% males; 61 ±7.7 yr  89% ever-smokers; 173 squamous cell, 152 adenocarcinoma;  Thoraxklinik Heidelberg-Rohrbach,  1996-1999 | 237/388  (61.1%) | *n*=351; 61% males; 55.2±14 yr;  67% ever-smokers; patients  from same hospital, no history  of malignancies, 1996-1999 | 196/345  (56.8%) | 481, 590, 803, 857 | blood samples;  PCR-RFLP, FRET Light Cycler; unconditional multivariate logistic  regression analyses adjusted  (age, gender, smoking status,  occupational exposure), Wald's test,  OR 95% CI | No, but higher risk with combined *NAT1* fast *-NAT2* slow genotypes |
|  |  |  |  |  |  |  |  |  |
| Zhou et al.  2002, USA | Caucasians | *n=*1115; 45.5% males;  65±10.8(26-91 yr); cumulative smoking exposure; patients with histologically  confirmed incident lung cancers,  smoking status; Massachusetts  General Hospital, 1992-2000 | 671/1115  (60.2%) | *n=*1250; 53.2% males;  58.5±12.3(19-100 yr); cumulative smoking exposure; friends and nonblood related family members of the lung cancer cases or  friends and nonblood  related family members of nonlung cancer patients at the cardiothoracic wards, smoking status | 750/1250  60.0%) | 481, 590,  803, 857 | blood samples;  PCR-RFLP; logistic regression and GAM adjusted (age, gender, smoking status, pack-years of smoking) OR 95% CI, S-plus , SAS | No, but significant interaction between *NAT2* genotype and pack-years of smoking;  rapid acetylator protective in non-smokers, but risk factors in heavy smokers;  Combined  *mEH-NAT2*  consistent with additive  effects modified by  smoking history |
|  |  |  |  |  |  |  |  |  |
| Skuladottir et al.  2005, Denmark,  Norway | Danish, Norwegian  Caucasians | *n=*320; 54% male; 20% <50 yr; 93% ever smokers; pooled data from 3 studies | 154/241  (63.9%) | *n=*618; age and gender matched with cases; 61% ever smokers; pooled data from 3 studies | 321/540  (59.4%) | pooled data | blood samples;  pooled data; conditional logistic regression, OR 95% CI, adjusted (age, gender, smoking status) χ^2^-test, STATA | No, but decreased risk rapid acetylators in ever smokers  and lower level duration  of smoking |
|  |  |  |  |  |  |  |  |  |
| Belogubova et al. 2005, Russia, Germany, Finland | Russian  Caucasians | *n=*178; 88% males; 60±10(29-84 yr);  85% smokers; 91 squamous cell; 46 adenocarcinoma, 26 small cell,  15 others; N.N. Petrov Institute  of Oncology (St-Petersburg) | 99/178  (55.6%) | *n=*364 middle-aged; 33% males; 36±10 (18-55 yr); 62% smokers; blood donations from same institute  *n=*351 elderly; 60% males;  50% smokers; 79± 4(75-95 yr);  tumor-free controls from same  institute | middle-aged:  218/364 (59.9%)  elderly:  208/351  (59.3%) | 282, 341,481, 590, 803, 857 | blood samples;  PCR-RFLP;  comparison of extremes approach;  χ^2^-test, crude odds ratios 95% CI, adjusted ORs  (gender, smoking status) Mantel-Haenszel method | No, even if smoking  history, gender, age or  lung cancer histology  considered |
|  |  |  |  |  |  |  |  |  |
| Habalova et al.  2005, Slovakia | Slovak,  Caucasians | *n=*110; 79% males; 92% smokers;  <60 yr 41%, >60 yr 59%;  75 squamous cell; Clinic of  Pneumology and Phtiseology  (Teaching Hospital Kosice) | 62/110  (56.4%) | *n=*167; 50% males; 26% smokers;  <60 yr 47%, >60 yr 53%; controls  without chronic respiratory/cancer  history at Teaching Hospital Kosice  (<65 yr Clinic of Hematology and  Blood Transfusion; >65 yr Geriatric  Centrum) | 87/167  (52.1%) | 341, 481, 590, 803, 857 | blood samples;  PCR-RFLP; Fisher's exact test,  Gart's OR 95% CI, adjusted ORs (age, gender, smoking status)  Arcus Quickstat Biomedical 1.1 | No, but genotype *NAT2*5B/*6* frequent  in younger, non-smokers with squamous cell carcinoma |
|  |  |  |  |  |  |  |  |  |
| Chiou et al.  2005, Taiwan | Chinese  (Asians) | *n=*162; 33% males; 63.2±11.4 yr;  never-smoking; 126 adenocarcinoma,  34 squamous cell, 2 small cell  carcinoma; Veterans General  Hospital-Taichung and Chung Shan  Medical University Hospital | 27/162  (16.7%) | *n=*208; 27 % males; 53.7±10.9 yr;  never-smoking with no history of  cancer; collected from community  health survey | 64/208  (30.8%) | 191, 481, 590, 857 | blood samples;  PCR-RFLP; χ^2^-test, OR 95% CI adjusted for age, gender, lung cancer histology  Multiple regression model SPSS  10.0 | Yes, increased risk with  rapid acetylators in female  never smokers; higher risk  with combined *CYP1A2*  fast-*NAT2* fast in female  never smokers |
|  |  |  |  |  |  |  |  |  |
| Sorensen et al.  2005, Denmark | Danish  Caucasians | *n=* 256; 50-65 yr; 84% smokers;  small cell (20%), adenocarcinoma (33%), squamous cell (22%);  population-based, 1994-2001 | 156/255  (61.2%) | *n=*269; 50-65 yr; 74.7% smokers;  population-based | 43/264  (54.2%) | 481,590, 803, 857 | blood samples,  sequencing and TaqMan assay  (481, 590), PCR-RFLP;  unweighted case-cohort approach,  rate ratios (RR) by Cox  proportional hazards models,  95% CI | No, but fast acetylator  protective in light but not  in heavy smokers |
